# *Stenotrophomonas maltophilia *prosthetic valve endocarditis: a case report

**DOI:** 10.1186/1752-1947-2-174

**Published:** 2008-05-23

**Authors:** Sophie Bayle, Clarisse Rovery, Pascal Sbragia, Didier Raoult, Philippe Brouqui

**Affiliations:** 1 Service des maladies infectieuses et tropicales, Hôpital Nord, Chemin des Bourrelys, 13915 Marseille cedex 20, France; 2 Service de cardiologie, Hôpital Nord, Chemin des Bourrelys, 13915 Marseille cedex 20, France; 3 Service de Microbiologie, Hôpital de la Timone, Marseille, France

## Abstract

**Introduction:**

*Stenotrophomonas maltophilia *is an environmental bacterium increasingly involved in nosocomial infections and resistant to most antibiotics. It is important to recognize and efficiently treat infections with this bacterium as soon as possible.

**Case presentation:**

We present a case of *Stenotrophomonas maltophilia *prosthetic valve endocarditis secondary to an indwelling catheter infection. The patient was cured without surgery. We review other cases of *S. maltophilia *endocarditis from the literature and describe the peculiarities of this case.

**Conclusion:**

*S. maltophilia *endocarditis is a rare disease that is often hospital-acquired and related to an indwelling catheter infection. The high lethality is likely related to the intrinsic resistance of nosocomial bloodstream infections to commonly prescribed antibiotics.

## Introduction

*Stenotrophomonas maltophilia *is a Gram-negative aerobic bacillus widely distributed in natural and human environments and has become increasingly responsible for nosocomial infections such as bacteremia, pneumonia, urinary tract infections, skin and soft tissue infections, ocular infections and meningitis [1]. It is generally considered as an opportunist pathogen with potential risk factors being: malignancies, debilitating illnesses, prior therapy with broad-spectrum antibiotics, chronic respiratory diseases, especially cystic fibrosis, prolonged endotracheal intubation and indwelling vascular catheters. *S. maltophilia *infective endocarditis (IE) is a rare and poorly described disease [2-5]. Here, we present the 29th reported case of *S. maltophilia *endocarditis along with a literature review.

## Case presentation

In November 2005, a 34-year-old Algerian woman was admitted to the infectious disease department for a suspected prosthetic mitral valve IE. She had a medical history of acute rheumatic fever when she was 10 years old. She had a mitral valve replacement with a prosthetic mechanic valve eight years ago. She was hospitalized at the end of a recent pregnancy because she needed intravenous heparin therapy in relay of oral anticoagulant for prevention of thrombosis of the mechanical valve prosthesis. Three days after delivery, she presented with a fever and peripheral catheter-related infection. The catheter was in place for six days; it was inserted at the admission of the patient to the high-risk pregnancy unit. The site of the catheter insertion appeared inflammatory and indurate. All three blood cultures (automated blood culture BACTEC 9240 system, Becton Dickinson, Le pont de Claix, France) grew on day 2 with *S. maltophilia*. Transoephageal echocardiography (TE) performed one day after the appearance of fever showed two small vegetations on the mitral mechanic valve without dehiscence. Based on the criteria established by Duke, the diagnosis of prosthetic mitral valve IE caused by *S. maltophilia *was confirmed. She was first treated empirically with vancomycin and gentamicin for 24 h. Antibiotics were then modified when we discovered Gram-negative bacillus growing in the blood cultures: imipenem and gentamicin were administered. She was treated with trimethoprim-sulfamethoxazole (SXT, trimethoprim (TMP) 4800 mg/day and sulfamethoxazole (SMZ) 960 mg/day) and gentamicin 3 mg/kg/day intravenously in accordance with antimicrobial susceptibility. In fact, effective treatment was started two days after the onset of disease and she had no fever on day 2 of this treatment. No cardiac complications occurred. Gentamicin was discontinued after seven days. SXT was then given *per os *(TMP 4800 mg/day and SMZ 960 mg/day). On day 8, a TE showed no vegetation. Antibiotics were continued for a total duration of six weeks. She remained well and showed no signs of fever and had a normal TE at her three-month follow-up visit.

## Discussion

Here we present the 29th reported case of *S. maltophilia *endocarditis. *S. maltophilia *IE is a rare disease [1-5]. In 2002, Crum et al reported one case of *S. maltophilia *IE and reviewed 24 additional cases [3]. A Medline search revealed three additional cases of *S. maltophilia *IE [2,4,5]. The characteristics of reported cases of *S. maltophilia *IE are summarized in additional files 1 [see additional file 1]. Underlying risk factors were found in all patients (27/27) including 8/27 (29.6%) of intravenous drug users (IVDUs). Among *S. maltophilia *IE, 14/27 (51.94%) were of nosocomial origins (4/14, 28.6% being catheter-related infections and 10/14, 71.4% related to recent valve replacement (< 9 months) whereas 13/27 (48.1%) were community-acquired (8/13, 61.5% being acquired in IVDUs).*S. maltophilia *is intrinsically resistant to many common antibiotics and broad-spectrum β-lactams, such as imipenem. Combination antibiotherapy is indicated with SXT as the agent of choice [1-6]. Therapy for *S. maltophilia *infections is problematic because of the broad resistance to antibiotics that is characteristic of this organism. Antibiotic treatments currently used for nosocomial primary bloodstream infections [7], such as vancomycin and imipenem, have no effect on *S. maltophilia*. This may explain the high mortality reported (9/28, 32%) and the frequency of complications (17/25, 68%) in *S. maltophilia *IE. Surgery is necessary in 9/17 (52.9%) cases involving prosthetic valves. Indeed, only 6/17 (35.3%) of *S. maltophilia *prosthetic valve IE are cured without surgery.

*S. maltophilia *endocarditis is a rare and severe disease, often nosocomial and related to central indwelling catheter infection. Our case is unusual as IE was secondary to peripheral catheter-related infection. As *S. Maltophilia *is resistant to the first line of antibiotic treatment used in these cases, empiric antibiotic treatment is often ineffective. Delay in effective antibiotic treatment is a major risk factor for mortality in *S. Maltophilia *bacteremia [8]. As a consequence, it is important to identify this microorganism as quickly as possible. Standard bacterial identification and susceptibility testing is normally a 48-h process. Polymerase chain reaction (PCR) can be used to decrease the identification time for Gram-negative bacteria isolated from blood culture, which has also been suggested for *Pseudomonas aeruginosa *infections [9]. In the light of this case report, it is also important to recall that guidelines on the prevention of intravascular device-related bloodstream infection have to be generalized and respected [10]. In fact, the catheter was left in place for too long in this previously healthy woman, and nosocomial infection could have easily been prevented in this patient.

## Conclusion

*S. maltophilia *is a rare and severe disease, and is usually hospital-acquired. It is important to identify this microorganism as quickly as possible since *S. maltophilia *is resistant to first line antibiotic therapy used in case of nosocomial infections. This case report also emphasizes that prevention of intravascular device-related bloodstream infection is a major measure to prevent this very severe disease.

## Competing interests

The authors declare that they have no competing interests.

## Authors' contributions

SB, CR, PB and DR contributed to the clinical work-up of the patient and writing-up of the manuscript. PS was in charge of the patient in the department of cardiology and performed the echocardiography. All authors read and approved the final manuscript.

## Consent

Written informed consent was obtained from the patient for publication of this case report and accompanying images. A copy of the written consent is available for review by the Editor-in-Chief of this journal.

## Supplementary Material

Additional file 1 Stenotrophomonas maltophilia infectious endocarditis case reports in the literrature. The data provided represent the Stenotrophomonas maltophilia infectious endocarditis cases reported in the literrature. Click here for file
