# Supplementary material for: Stenotrophomonas maltophilia prosthetic valve endocarditis: a case report
Source: J Med Case Rep. 2008 May 23;2:174. doi: 10.1186/1752-1947-2-174 (PMC2432066; doi:10.1186/1752-1947-2-174)
Supplement: Supplementary file 1 [file 1752-1947-2-174-S1.doc]

Table 1 - Summary of Stenotrophomonas maltophilia infectious endocarditis data
Abbreviations: AMC, ampicillin; AMK, amikacin; ASD, atrial septal defect; CAR, carbenicillin; CAZ, ceftazidime; CHF, congestive heart failure; CHL, chloramphenicol; CIP, ciprofloxacin; COL, colistin; CVC, central venous catheter; FEP, cefepime; GEN, gentamicin; IVDU, intravenous drug user; KAN, kanamycin; LVX, levofloxacin; MI, myocardial infarction; MOX, moxalactam; NAV, natural aortic valve; NMV, natural mitral valve; NR, not reported; PAV, prosthetic aortic valve; PEN, penicillin; PMV, prosthetic mitral valve; POL, polymyxin; PR, present report; STR, streptomycin; SXT, trimethoprim-sulfamethoxazole; TIC, ticarcillin; TIM = ticarcillin-clavulanic acid; TOB, tobramycin; TZP, piperacillin-tazobactam; VAN, vancomycin; VSD, ventricular septal defect.

Case	
Reference	Age, sex	Predisposing factors	Valve involved	Treatment	Surgery	Complications	Outcome		1	[3]	26, M	Recent valve replacement (< 1 month)	PMV, ASD patch	CHL, KAN, COL	Yes	Multiple septic emboli	Died		2	[3]	30, M	Recent valve replacement (< 1 month)	PMV, ASD patch	CHL	No	None	Cured		3	[3]	65, F	Cystoscopy, valve replacement (7 months)	PMV	CAR, GEN, KAN, CHL, PEN, POL 	Yes	Persistent bacteremia	Cured		4	[3]	35, M	Recent valve replacement (early), rheumatic carditis	PMV	CAR, GEN, SXT	No	None	Cured		5	[3]	38, M	None	PMV	STR, PEN	No	septic emboli, MI	Died		6	[3]	22, M	IVDU	PAV	CAR, AMK, SXT	Yes	CHF, septic emboli	Cured		7	[3]	31, F	IVDU, dental treatment	PAV	CAR, KAN, SXT	Yes	Perivalvular abscess	Cured		8	[3]	57, M	IVDU, rheumatic carditis	NAV, NMV	POL, SXT	No	Septic emboli	Cured		9	[3]	25, M	None	VSD repair	GEN, CHL	No	NR	Died		10	[3]	33, M	IVDU, aortic stenosis, atrial fistula	NAV, NTV	TIC, MOX, SXT	Yes	CHF, myocardial abscess	Died		11	[3]	NR	CVC	NTV	NR	NR	NR	Died		12	[3]	NR	NR	NAV	NR	NR	NR	Died		13	[3]	NR	CVC	NAV	NR	NR	NR	Cured		14	[3]	33, M	IVDU, dental treatment	PAV	SXT, AMC, GEN	Yes	Perivalvular abscess	Cured		15	[3]	56, M	Recent valve replacement (early)	PAV 	CAZ, GEN, SXT	Yes	Septic emboli	Died		16	[3]	32, M	IVDU, subcutaneous reservoir	NTV	SXT	No	CHF	Died		17	[3]	28, M	IVDU	NAV	CIP, GEN	Yes	Myocardial abscess	Cured		18	[3]	60, F	Ventriculo-atrial shunt	NTV	TIM, SXT	No	Lung abscess	Cured		19	[3]	36, M	Dental treatment (3 months)	NAV	TZP, GEN	Yes	CHF	Cured		20	[3]	69, F	Recent valve replacement (3 months)	PMV, PAV	CAZ, GEN, CIP, SXT	Yes	CHF, persistent bacteremia	Died		21	[8]	37, M	Recent mitral valvuloplasty (early)	PMV	CAZ, AMK, CIP 
then TIM, SXT, COL	Yes	None	Cured		22	[3]	58, F	Recent valve replacement (3 months)	PMV	SXT	Yes	None	Cured		23	[3]	62, M	Recent valve replacement (6 months)	PAV	CIP, CHL	No	Aortic dissection	Cured		24	[3]	40, M	Recent valve replacement (9 months)	PAV	TIM, SXT	No	None	Cured		25	[3]	44, M	Rheumatic valvular disease	PMV	VAN, GEN	No	Recurrence with septic emboli treated by TMP-SMZ + TOB and surgery	Cured		26	[5]	44, M	IVDU, HIV, CHV, BHV, dental treatment, rheumatic aortic and mitral disease	NMV, NAV	LVX, SXT	No	None	Cured		27	[3]	56, F	CVC	NAV	FEP, CIP, SXT	No	None	Cured		28	[7]	65, F	NR	PAV	NR	NR	CHF, paravalvular abscess	NR		29	PR	34, F	Peripheral catheter	PMV	SXT, GEN	No	None	Cured
